# Purification of ribosomes from human embryonic stem (hES) cells for high-resolution Cryo-EM structural studies

**DOI:** 10.3389/fmolb.2026.1778824

**Published:** 2026-02-19

**Authors:** Disha-Gajanan Hiregange, Aliza Fedorenko, Elena Ainbinder, Anat Bashan, Ada Yonath

**Affiliations:** 1 Department of Chemical and Structural Biology, Weizmann Institute of Science, Rehovot, Israel; 2 Department of Life Science Core Facilities, Weizmann Institute of Science, Rehovot, Israel

**Keywords:** cryo-EM, hESC, ribosome, SPA, structure

## Abstract

Human ribosome structures at near-atomic resolution have been determined predominantly from transformed cell lines, often using translation inhibitors during purification that can introduce structural artifacts. We present a robust and scalable protocol for purifying 80S ribosomes from human embryonic stem (hES) cell cultures. The method addresses two common bottlenecks for structural studies of ribosomes from pluripotent cells: limited starting material and structural perturbations introduced by translation inhibitors such as cycloheximide or anisomycin. By combining gentle lysis and rapid clarification with a sucrose-cushion concentration step followed by gradient based purification the workflow preserves native ribosome conformations and yields highly pure 80S ribosomes suitable for single-particle cryo-EM at near-atomic resolution model building. The workflow was reproduced across independent preparations with consistent yields and map quality. As expected for inhibitor-free purification, no density corresponding to elongation inhibitors was observed in ribosomal functional centers. We supply a detailed, scalable protocol that includes buffer recipes, expected yields, quality control and troubleshooting steps, and recommendations for cryo-EM grid preparation. The procedure is transferable to other sensitive cell types (for example, primary B cells and iPS cells). This workflow expands access to native human ribosomes from non-transformed, developmentally relevant cells for structural and mechanistic studies of translation.

## Introduction

Ribosomes are essential macromolecular machines that catalyse protein synthesis in all living cells and play a central role in regulating gene expression at the translational level (Noller, 2024). In eukaryotes, ribosomes are large, dynamic ribonucleoprotein assemblies composed of four ribosomal RNAs and approximately 80 ribosomal proteins, whose structural organization underpins translation initiation, elongation, termination, and quality control (Ben-Shem et al., 2011; Klinge and Woolford, 2019; Cruz et al., 2015; Hinnebusch, 2014). Over the past decade, advances in single-particle cryo-electron microscopy (cryo-EM) have enabled near-atomic resolution reconstructions of eukaryotic ribosomes, revealing detailed molecular mechanisms of translation and its regulation in health and disease (Shalev-Benami et al., 2016; Hiregange et al., 2022; James and Smyth, 2018; Matzov et al., 2020; Lomakin and Steitz, 2013; Natchiar et al., 2017; Gemmer et al., 2023; Ameismeier et al., 2018).

High-resolution structures of the human ribosome have primarily been obtained from transformed or cancer-derived cell lines, most notably HeLa cells, owing to their robustness, scalability, and tolerance to extensive biochemical manipulation (Holvec et al., 2024; Anger et al., 2013; Khatter et al., 2015; Myasnikov et al., 2016; Natchiar et al., 2017; Behrmann et al., 2015; Voorhees and Ramakrishnan, 2013; Belin et al., 2010). While these systems have been invaluable, they may not fully capture the native translational landscape of non-transformed cells. Increasing evidence suggests that ribosome composition, rRNA modification patterns, and association with regulatory factors can vary across cell types, developmental stages, and physiological states, giving rise to functionally specialized ribosomes (Norris et al., 2021; Shi et al., 2017; Xue and Barna, 2012; Genuth and Barna, 2018; Simsek et al., 2017; Slavov et al., 2015; Nachtergaele and He, 2017). Structural characterization of ribosomes from developmentally naïve and non-transformed human cells is therefore essential to understand how translation is regulated in early human development.

Human embryonic stem (hES) cells provide a powerful model for studying translational control in pluripotency and early lineage commitment. Pluripotent cells exhibit distinct translational programs characterized by tight regulation of protein synthesis rates, selective translation of specific mRNAs, and dynamic remodelling of ribosome biogenesis pathways (Gabut et al., 2020; Häfner et al., 2023; Ingolia et al., 2011; Sampath et al., 2008; Genuth et al., 2022). Perturbations in ribosome production or function have been shown to directly impact pluripotency maintenance and differentiation potential. Notably, human embryonic stem cells (hESCs) display distinct nucleolar morphology and dynamic ribosome biogenesis compared to transformed cell lines such as HeLa or HEK293, reflecting a tightly regulated “balancing act” that ensures proper protein synthesis during development (Lafontaine et al., 2021; Correll et al., 2019). Disruption of this regulation is a common feature of cancer-derived lines, where nucleolar architecture and ribosome production are often uncoupled from translational needs. Understanding how ribosome composition and biogenesis are controlled in physiologically relevant, non-transformed systems is therefore essential for dissecting the principles of selective translation and its role in development and disease. Despite this biological relevance, ribosomes derived from hES cells remain largely unexplored at the structural level, in part due to technical challenges associated with their handling and sensitivity during their purification.

Purification of intact ribosomes from hES cells is complicated owing to several factors, including limited availability of starting material, heightened sensitivity to stress, and the need to preserve native ribosomal conformations for structural analysis. Most established ribosome purification protocols rely on translation inhibitors such as cycloheximide, anisomycin, or harringtonine to arrest elongation and prevent ribosome run-off during cell lysis (Duncan and Mata, 2017; Ingolia et al., 2009; Gerashchenko and Gladyshev, 2014; Hussmann et al., 2015). Although effective for stabilizing translating ribosomes, these compounds bind directly to conserved functional sites on the ribosome and can induce conformational changes, alter ligand occupancy, or obscure physiologically relevant structural states (Budkevich et al., 2014; Holm et al., 2023; Behrmann et al., 2015; Gerashchenko and Gladyshev, 2014). Such effects are particularly problematic for high-resolution cryo-EM studies that aim to capture native ribosome conformations.

Developing antibiotic-free ribosome purification strategies is therefore critical for structural studies that seek to avoid drug or induced artefacts by non-ribosomal cellular compounds. However, removal of translation inhibitors necessitates careful optimization of lysis conditions, ionic composition, and processing speed to minimize ribosome dissociation and RNA degradation. These challenges are particularly pronounced in sensitive cell types, such as hES cells, for which standard protocols developed in yeast or immortalized mammalian cell lines are often poorly suited.

Here, we present a robust and scalable protocol for purifying native 80S ribosomes from human embryonic stem cells without the use of translation antibiotics. The method combines gentle detergent-based lysis, rapid clarification, sucrose-cushion concentration, and density gradient polishing under optimized ionic conditions to preserve ribosome integrity and homogeneity. Importantly, this workflow yields ribosomal material suitable for high-resolution single-particle cryo-EM analysis and is transferable to other sensitive mammalian cell types, including primary B cells and induced pluripotent stem (iPS) cells. To our knowledge, this protocol enables the first high-resolution structural analysis of human ribosomes derived from embryonic stem cells, providing a foundation for future studies of translational regulation in human development.

## Materials, reagents, and equipment

### Cell lines and biological materials

- Human embryonic stem cell line H9 (WA-09; WiCell, Madison, WI)
- Irradiated mouse embryonic fibroblasts (iMEFs; Stem Cell Core, Weizmann Institute of Science)
- GelTrex matrix (Gibco)

### Cell culture reagents

- DMEM/F-12 (Sigma-Aldrich)
- KnockOut Serum Replacement (KOSR; Gibco)
- GlutaMAX supplement (200 mM; Gibco)
- MEM Non-Essential Amino Acids Solution (100×; Sartorius)
- β-Mercaptoethanol (Gibco)
- Recombinant human FGF-2 (PeproTech)
- StemFlex medium (Gibco)
- Collagenase IV (Worthington)
- Phosphate-buffered saline (PBS), Ca^2+^/Mg^2+^-free

### Chemicals and reagents for ribosome purification

- Tris base and Tris-HCl
- HEPES
- Sodium chloride (NaCl)
- Potassium acetate (KOAc)
- Magnesium chloride (MgCl_2_)
- Magnesium acetate [Mg(OAc)_2_]
- Ammonium acetate (NH_4_OAc)
- Dithiothreitol (DTT)
- Triton X-100
- Sucrose (ultrapure, molecular biology grade)
- RNasin ribonuclease inhibitor (Promega)
- cOmplete Mini EDTA-free protease inhibitor cocktail (Roche)
- Bentonite (RNase-adsorbing grade)

### Electron microscopy reagents

- Uranyl acetate (2% w/v solution)
- Milli-Q or equivalent ultrapure water
- Carbon-coated copper EM grids

### Consumables

- 10-cm tissue culture dishes
- 50-mL and 15-mL conical tubes
- Ultracentrifuge tubes compatible with Ti70, SW-28 and TLA 100.2 rotors
- Filter paper (Whatman)
- Parafilm
- Pipette tips (RNase-free)
- 0.2 µM filter apparatus

### Equipment

- Tissue culture incubator (37 °C, 5% CO_2_)
- Refrigerated tabletop centrifuge
- Ultracentrifuge (Beckman Coulter or equivalent)
- SW-28 swinging-bucket rotor (Beckman Coulter)
- Ti70 rotor (Beckman Coulter)
- TLA 100.2 fixed-angle rotor (Beckman Coulter)
- Gradient maker or gradient-forming system
- Gradient fractionation system with UV detector (1-mm pathlength)
- UV-Vis spectrophotometer for A260 measurements
- Glow discharge unit for EM grids
- Transmission electron microscope for negative staining

### Formulation of solutions and buffers

All solutions were prepared using RNase-free water and analytical-grade reagents. Buffers were stored at 4 °C unless otherwise indicated. DTT, RNase inhibitors, and protease inhibitors were added fresh immediately before use. Sucrose solutions were treated with bentonite after preparation to minimize RNase contamination (Jacoli et al., 1973). Bentonite was added to the buffer at a concentration of ∼10 mg/mL and mixed thoroughly by gentle stirring (sometimes heated if needed). The mixture was incubated at room temperature for ∼10–20 min to allow RNases to adsorb onto the clay. Following incubation, the bentonite was removed by filtering using 0.2 µM filter apparatus.

### Rationale for buffer composition and processing conditions

Ionic conditions and processing speed were optimized empirically to preserve intact 80S ribosomes from sensitive human cell types in the absence of translation inhibitors. Magnesium acetate (10 mM) was required to minimize subunit dissociation during lysis and ultracentrifugation. In contrast, lower magnesium concentrations resulted in partial 60S-40S separation as assessed by sucrose gradient profiles and negative-stain EM. Potassium acetate concentrations between 100–150 mM provided optimal ribosome stability while limiting aggregation and non-specific association of cellular components. All purification steps were performed at 4 °C with minimal handling time; delays of >∼30 min between lysis and ultracentrifugation reproducibly led to degradation and reduced ribosome yield.

#### hESb medium

DMEM/F-12
20% (v/v) KnockOut Serum Replacement
2 mM GlutaMAX
1× MEM non-essential amino acids
100 μM β-mercaptoethanol
8 ng/mL recombinant human FGF-2

#### Lysis buffer A

30 mM Tris-HCl, pH 7.5
300 mM NaCl
20 mM MgCl_2_
2% (v/v) Triton X-100
RNasin (as recommended by manufacturer)
1× cOmplete Mini EDTA-free protease inhibitor cocktail

#### Buffer B (sucrose cushion buffer)

45 mM HEPES-KOH, pH 7.5
150 mM potassium acetate (KOAc)
10 mM magnesium acetate [Mg(OAc)_2_]
2 mM DTT

#### Sucrose cushion (1.1 M)

1.1 M sucrose prepared in buffer B

#### Buffer C (gradient and resuspension buffer)

20 mM Tris-HCl, pH 7.5
150 mM potassium acetate (KOAc)
10 mM magnesium acetate [Mg(OAc)_2_]
2 mM DTT
RNasin (added fresh)

#### Sucrose gradients (10%–40% w/v)

10%–40% (w/v) sucrose prepared in buffer C

#### Buffer D (final storage buffer)

20 mM Tris-HCl, pH 7.5
100 mM potassium acetate (KOAc)
10 mM magnesium acetate [Mg(OAc)_2_]
10 mM ammonium acetate (NH_4_OAc)
1 mM DTT

#### Negative stain solution

2% (w/v) uranyl acetate in water (filtered immediately before use)

## Methods

### Objectives of the method

The objective of this method is to purify intact, native 80S ribosomes from hES cells without the use of translation antibiotics, while maintaining sufficient yield and homogeneity for high-resolution single-particle cryo-EM, while preserving *native* ribosome conformations. The protocol is designed to (i) minimize structural perturbations associated with translation inhibitors, (ii) accommodate the limited and sensitive nature of hES cells starting material, and (iii) be scalable and transferable to other sensitive mammalian cell types. A secondary objective is to provide a reproducible workflow with clearly defined quality-control checkpoints that enable reliable downstream structural analysis.

### Validation of the method

Method validation was achieved through multiple complementary readouts. Ribosome integrity and purity were assessed by sucrose density gradient profiles (A260 absorbance), which consistently revealed discrete ribosomal peaks with minimal background. Negative-stain electron microscopy demonstrated monodisperse particles with canonical ribosome morphology and low aggregation. Purified ribosomes were subsequently used for single-particle cryo-EM, yielding near-atomic resolution reconstructions under antibiotic-free conditions, thereby confirming structural suitability. The transferability of the protocol was validated by successful application to other sensitive mammalian cell types, including primary B cells, and iPS cells, without modification of buffer composition or centrifugation parameters. In addition, the workflow is compatible with downstream reconstitution under native conditions. Using the same protocol, ribosomes from HEK293 cells were purified and assembled into ribosome-mRNA complexes. Structural analyses of these assemblies will be reported in a separate, forthcoming manuscript. Similarly, ribosomes purified from primary B cells using this protocol will be included in an independent upcoming study. All preparations were independently reproduced at least three times, yielding consistent ribosome integrity, particle quality, and map resolution.

### Step-by-step procedure

#### Step 1. cell culture of hES cells

The hES cell line H9 (WA-09; WiCell, Madison, WI) was maintained in an undifferentiated state on irradiated mouse embryonic fibroblasts (iMEFs; Stem Cell Core, Weizmann Institute of Science) using hESb medium. Cells were passaged using Collagenase IV (Worthington).

To eliminate iMEFs prior to ribosome purification, hES cells were transferred to GelTrex-coated tissue culture plates (Gibco) and cultured in StemFlex medium (Gibco) for two to three passages. All cultures were maintained without translation inhibitors or antibiotics.

#### Step 2. cell preparation and harvest

##### Timing: 1–1.5 h

1. For ribosome purification, approximately 6 × 10^8 H9 cells were harvested from 39 × 10 cm dishes. Culture medium was removed, and cells were washed twice with ice-cold PBS.
2. Residual PBS was retained to prevent cell drying, and cells were released by gentle scraping on ice. Cells were pooled into 50 mL conical tubes and centrifugated at 200 *g* for 10 min at 4 °C.
3. Pellets were resuspended in ice-cold PBS and centrifuged again under the same conditions to remove residual medium.
4. After removal of PBS, pellets were resuspended in an equal volume of fresh ice-cold PBS (typically ∼2 mL total volume) and kept on ice for immediate lysis.

Pause point: Cell pellets may be flash-frozen in liquid nitrogen and stored at −80 °C for short periods; however, optimal results are obtained with freshly harvested cells.

Note: While our current protocol uses multiple 10-cm dishes, laboratories seeking higher throughput could consider multi-layer culture systems such as CellStacks, HYPERFlasks, or TripleFlasks to potentially reduce handling time and improve reproducibility. We emphasize, however, that the efficacy of these systems in an inhibitor-free ribosome purification workflow has not been tested and would require experimental validation.

#### Step 3. cell lysis and clarification

##### Timing: 30–40 min

1. Cells were lysed in freshly prepared lysis buffer A. RNasin ribonuclease inhibitor (Promega) and 1× cOmplete Mini EDTA-free protease inhibitor cocktail (Roche) were added immediately before use.
2. Cell suspensions were mixed gently by inversion and incubated on ice for 10 min to ensure complete lysis.
3. Lysates were clarified by centrifugation at 9,200 *g* for 10 min at 4 °C to remove nuclei and insoluble debris.
4. The clarified supernatant was carefully transferred to a new tube without disturbing the pellet, no pause points were introduced at this stage.

#### Step 4: ribosome pelleting through a sucrose cushion

##### Timing: ∼18 h (including overnight centrifugation)

1. Clarified lysates were first transferred into ultracentrifuge tubes. A 1.1 M sucrose cushion prepared in buffer B was then carefully underlaid beneath the lysate using a syringe fitted with a long needle, forming a sharp lysate-cushion interface prior to centrifugation. All sucrose solutions were treated with bentonite after preparation to inhibit ribonuclease activity. Delays between clarification and ultracentrifugation resulted in reduced recovery of intact 80S ribosomes and were therefore avoided.
2. Samples were centrifuged at 210,000 *g* (45,000 rpm) for 16 h at 4 °C in an ultracentrifuge by Ti70 rotor (Beckman Coulter) to pellet ribosomes. Following centrifugation, the supernatant was carefully removed, and buffer C was added. Ribosomal pellets were resuspended to homogeneity by gentle pipetting and incubation on ice.

To remove any incompletely resuspended material or aggregates, suspensions were centrifuged at 9,200 *g* for 10 min at 4 °C. Only the clarified supernatant was retained for subsequent purification steps. Ribosome concentration at this stage was monitored by absorbance at 260 nm; a typical preparation yielded approximately 30 A260 units in 600 μL.

Pause point: Resuspended ribosomes can be flash-frozen in liquid nitrogen and stored at −80 °C for several weeks prior to gradient loading.

#### Step 5: sucrose density gradient centrifugation

##### Timing: 12–14 h

1. Linear 10%–40% (w/v) sucrose gradients were prepared using a gradient maker in buffer C.
2. Gradient buffer conditions were optimized to maximize recovery of intact 80S ribosomes under inhibitor-free conditions rather than to achieve maximal separation of 40S and 60S subunits. Polysomes could also be recovered from the trailing shoulder of the monosome peak. Gradient solutions were treated with bentonite to inhibit RNase contamination and equilibrated to 4 °C prior to use.
3. Clarified ribosome preparations were layered onto the top of the gradients and centrifuged at 87,000 *g* (22,000 rpm) for 11 h at 4 °C in an SW-28 rotor (Beckman Coulter). This step enabled the separation of ribosomal subunits and monosomes and the removal of residual contaminants.

### Choice of centrifugation rotor

The SW-28 rotor was selected to accommodate the total lysate volume obtained from hES cell preparations and to ensure reproducible gradient formation and fractionation across experiments. While smaller rotors (e.g., SW-40 or SW-60) may reduce centrifugation times when processing lower volumes, the SW-28 provided consistent peak resolution and recovery during method establishment and transferability testing. The protocol is modular and can be adapted to alternative rotors depending on available equipment and scale.

#### Step 6: gradient fractionation and ribosome collection

##### Timing: 3–4 h

1. Following centrifugation, gradients were fractionated using an automated gradient fractionation system equipped with a 1-mm pathlength UV detector.
2. Gradients were pumped at 1.5 mL/min, and fractions of 0.75 mL were collected while continuously monitoring absorbance at 260 nm.
3. Major A260 peaks were consistently observed, corresponding to ribosomal subunits, 80S or polysomes. Fractions corresponding to each peak were pooled separately and concentrated by ultracentrifugation at 320,000 × g (56,000 rpm) for 16 h at 4 °C in Ti70 rotor (Beckman Coulter).

#### Step 7. final pelleting, buffer exchange, and storage

##### Timing: 16–20 h

1. Ribosomal pellets were resuspended in buffer D.
2. Samples were further centrifuged at 200,000 *g* (75,000 rpm) for 2.5 h at 4 °C using a TLA 100.2 rotor (Beckman Coulter) to remove residual sucrose and exchange buffer.
3. Final ribosomal pellets were gently dissolved in buffer D by overnight incubation at 4 °C without agitation.
4. Aliquot purified ribosomes, flash-freeze in liquid nitrogen, and store at −80 °C.

Pause point: Purified ribosomes can be stored at −80 °C for several months without detectable loss of integrity.

### Negative-stain electron microscopy

Purified ribosomes were assessed by negative-stain transmission electron microscopy prior to cryo-EM grid preparation. Samples were diluted to a final concentration of ∼0.2 mg/mL. Carbon-coated copper grids were glow-discharged immediately before use.

A 3.5-μL aliquot of sample was applied to the grid and incubated for 30 s, after which excess liquid was blotted with filter paper. Grids were briefly washed by touching the grid surface to drops of Milli-Q water and subsequently stained by sequential application of 2% (w/v) uranyl acetate. Each staining step was followed by blotting with filter paper. Grids were thoroughly air-dried before imaging. Clean tweezers were maintained by washing with ethanol between samples.

### Single particle Cryo-EM data collection and refinement for ribosomes from hES cells and B cells

Purified 80S ribosomes from hES cells and B cells were applied to glow-discharged holey carbon grids overlaid with a continuous thin carbon film. The grids were blotted and plunge-frozen using a Vitrobot Mark IV (Thermo-Fischer Scientific) under the following conditions: 3.5 μL sample volume, 2.5-s blot time, 30-s wait time, and blot force of −1. Cryo-EM micrographs were collected at liquid nitrogen temperature with a Titan Krios electron microscope (Thermo-Fischer Scientific) operating at 300 kV, equipped with a K3 direct electron detector (Gatan Inc.). For hES cells 80S ribosomes, the nominal magnification was 105K with a pixel size of 0.842 Å/pixel, and for B cells 80S ribosomes, a pixel size of 0.824 Å/pixel. Defocus values ranged from −0.5 to −1.5 µm. Data were processed using RELION 4.0 (Kimanius et al., 2021), with motion correction handled by MotionCor2 (Zheng et al., 2017) and contrast transfer function (CTF) parameters estimated via CTFFIND-3 (Mindell and Grigorieff, 2003). Initial 3D models were generated using semi-automatic particle picking and reference-free 2D classification. Unsupervised 3D classification was performed using a 60 Å low-pass filtered cryo-EM map as a reference, followed by refinement steps including CTF refinement, particle polishing, and 3D refinement to produce a high-resolution density map. Multi-body refinement cycles were subsequently applied to further improve the resolution (Nakane and Scheres, 2021).

## Results

### Expected outcomes of the protocol

**FIGURE 1:**
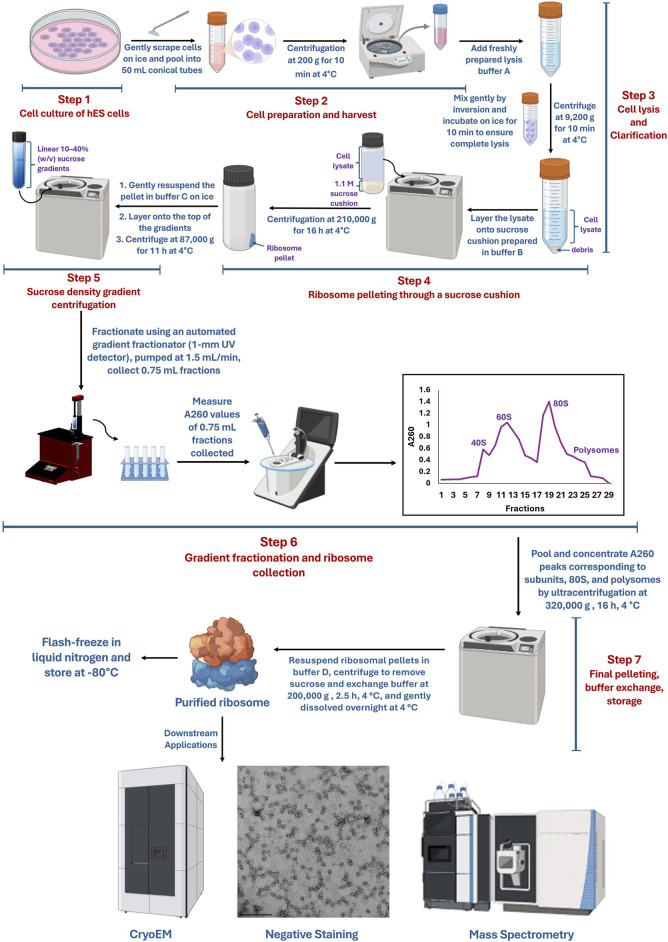
Workflow for the purification of human ribosomes from human embryonic stem cells. Created using BioRender. Pavlovic Djuranovic, S. (2025) https://BioRender.com/0uym2rp.

**FIGURE 2:**
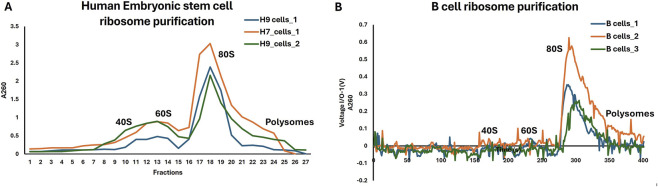
Ribosome purification by sucrose gradient. **(A)** hES cells (The Graphs were obtained from measurement of A260 value of fractions using nanophotometer) **(B)** B cells (The Graphs were obtained from MultiLab® by UV-Vis spectrometry detection at A260).

Application of the antibiotic-free purification workflow to hES cells reproducibly preserved *native* ribosome conformations homogeneous ribosomal particles suitable for high-resolution structural analysis. Starting from approximately 6 × 10^8^ H9 hES cells, sucrose cushion centrifugation produced ribosome-enriched pellets that could be efficiently resolved by sucrose density gradient ultracentrifugation. UV absorbance (A260) profiles consistently displayed discrete and reproducible ribosomal peaks corresponding to ribosomal species, with prominent enrichment of intact 80S ribosomes and minimal baseline signal, indicative of low contamination by non-ribosomal ribonucleoprotein complexes (Figure 1). Because gradients were fractionated and analyzed by discrete A260 measurements rather than continuous UV monitoring, peak shape should be interpreted qualitatively rather than as a high-resolution assessment of subunit heterogeneity. Ribosome integrity and homogeneity were further validated by negative-stain EM and by the ability to obtain near-atomic resolution cryo-EM reconstructions, which provide a more sensitive readout of sample quality than gradient peak shape alone. All preparations were independently biologically reproduced at least three times. Comparable gradient profiles were obtained from independent preparations and from other sensitive mammalian cell types, including primary B cells and iPS cells, demonstrating the robustness and reproducibility of the method across distinct cellular contexts (Figure 2).

### Assessment of ribosome integrity by negative-stain electron microscopy

**FIGURE 3:**
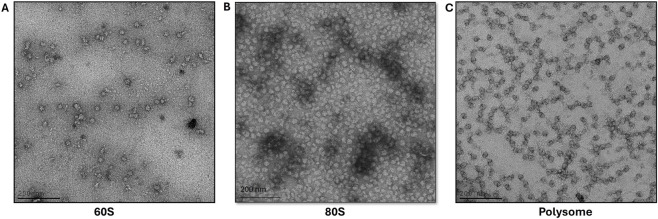
Negative staining images of the ribosome samples obtained from hES cells ribosome purification. **(A)** 60S subunits **(B)** 80S ribosomes and **(C)** Polysome. Scale bars are left-bottom (200 nm).

Negative-stain electron microscopy of purified ribosomes revealed monodisperse particles with well-defined ribosomal morphology and minimal aggregation (Figure 3). Individual particles exhibited characteristic size and shape consistent with intact eukaryotic 80S ribosomes, and no evidence of large-scale dissociation, degradation, or aggregation was observed. These features are predictive of successful vitrification and high-quality cryo-EM data collection, validating that the purification preserves ribosomal integrity despite the absence of translation inhibitors.

### Cryo-EM map quality and resolution

**FIGURE 4:**
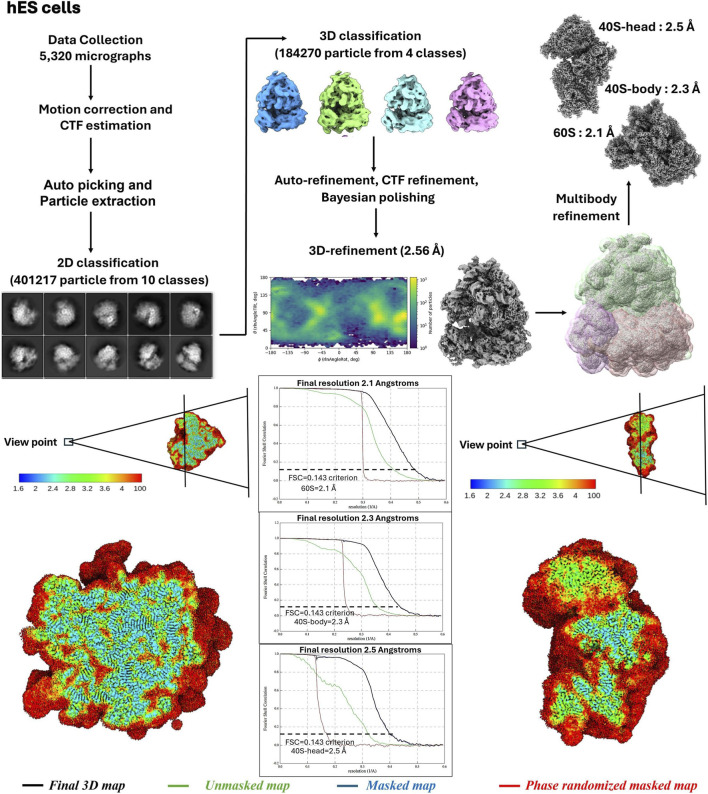
The cryo-EM image reconstruction flow chart for the hES cells ribosome and Local map resolution and Fourier Shell Correlation (FSC) curves for the ribosome. Cross-section rendering of large subunit (left) and small subunit (right) of the cryo-EM density maps of the ribosome, colored according to local resolution distribution. “Gold standard” FSC curves for the final 3D map (black), unmasked map (green), masked map (blue), and phase randomized masked map (red) indicate nominal resolutions of 60S is 2.1 Å, 40S-body 2.3 Å and 40S-head 2.5 Å for hES cells ribosome (FSC = 0.143 criterion) of the masked map.

**FIGURE 5:**
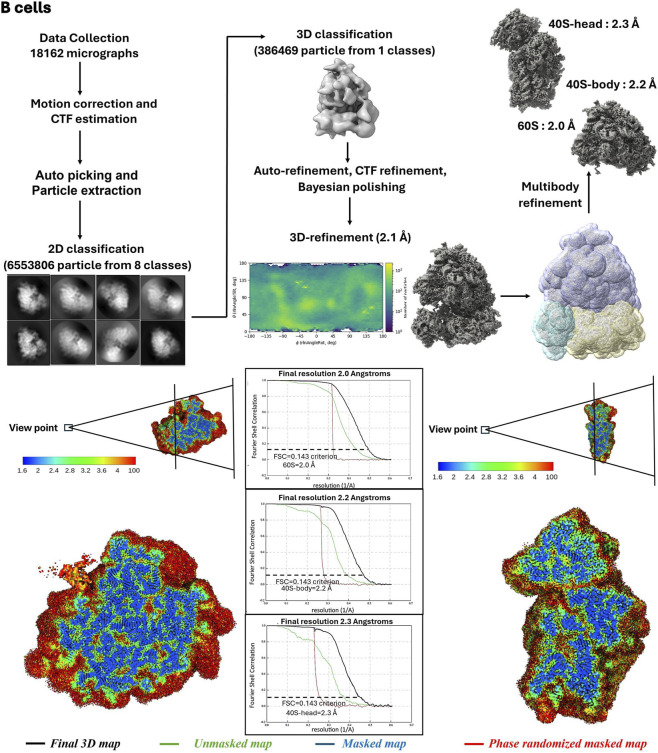
The cryo-EM image reconstruction flow chart for the B cells ribosome and Local map resolution and Fourier Shell Correlation (FSC) curves for the ribosome. Cross-section rendering of large subunit (left) and small subunit (right) of the cryo-EM density maps of the ribosome, colored according to local resolution distribution. “Gold standard” FSC curves for the final 3D map (black), unmasked map (green), masked map (blue), and phase randomized masked map (red) indicate nominal resolutions of 60S is 2.0 Å, 40S-body 2.2 Å and 40S-head 2.3 Å for B cells ribosome (FSC = 0.143 criterion) of the masked map.

Purified ribosomes from both hES cells and B cells supported single-particle cryo-EM analysis and yielded high-resolution reconstructions under native, antibiotic-free conditions (Figures 4, 5). Gold-standard Fourier shell correlation (FSC) analysis indicated near-atomic nominal resolutions, confirming that the biochemical purity and homogeneity achieved by the protocol are sufficient for detailed structural interpretation.

Local resolution analysis demonstrated that the ribosomal core, including rRNA-rich regions, decoding centre, and peptidyl transferase centre, reached higher local resolution than peripheral regions (Figures 4, 5). This distribution is consistent with well-behaved ribosome datasets and supports the reliability of the reported global resolutions.

### Translational state heterogeneity

**FIGURE 6:**
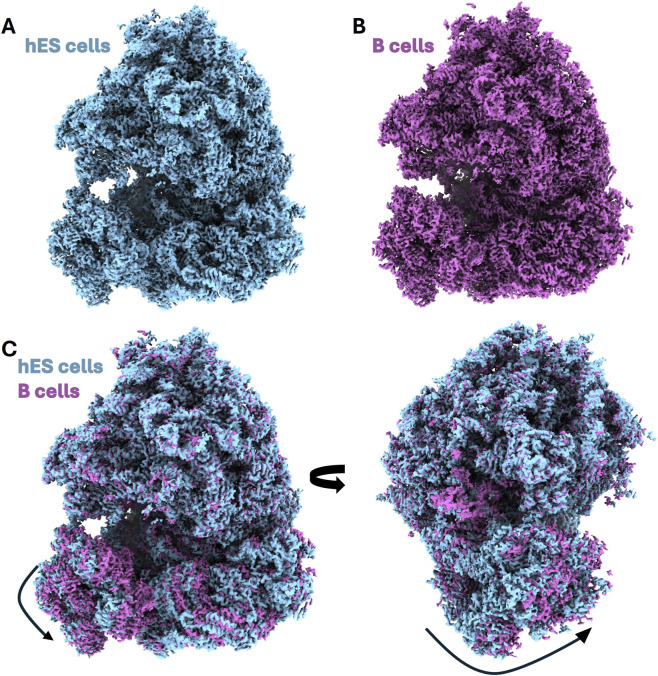
Conformational differences and rotational dynamics of human ribosomes. **(A)** hES cell 80S ribosome cryo-EM map from hES cells colored in light blue. **(B)** B cell 80S ribosome cryo-EM map of the 80S ribosome from B cells colored in pink. **(C)** Superposition and Rotational Analysis: Structural overlay of the hES cell (blue) and B cell (pink) cryo-EM ribosome maps. The black arrows indicate the intersubunit rotation of the small (40S) subunit relative to the large (60S) subunit. The side-by-side views in **(C)** illustrate the displacement between the two states, highlighting distinct global conformational signatures as depicted in the different cell types.

Because no translation inhibitors or stalling agents were used at any stage of purification, we assessed whether the recovered ribosomes retained native functional state diversity. 3D classification of hES cell derived 80S ribosomes revealed a largely homogeneous population adopting a non-rotated conformation and lacking well-resolved density for A-, or P-, site tRNAs or translation factors. Rotated or factor-bound states were not strongly populated. In contrast, density corresponding to an E-site tRNA was observed at the ribosome in the region where cycloheximide is known to bind. Importantly, applying the same purification strategy to other human cell types (i.e., B cells and iPS cells) resulted in distinct distributions of ribosomal states, indicating that translational state composition reflects cellular context rather than being imposed by the purification procedure (Figure 6). Consistent with sampling under native, inhibitor-free conditions, these results demonstrate that the method preserves ribosomal integrity without artificially stabilizing drug-arrested intermediates and provides an unbiased structural baseline that enables direct comparison of ribosomes across cell types and developmental states, as well as combination with defined perturbations when enrichment of specific translational states is desired.

### Model building and validation

**FIGURE 7:**
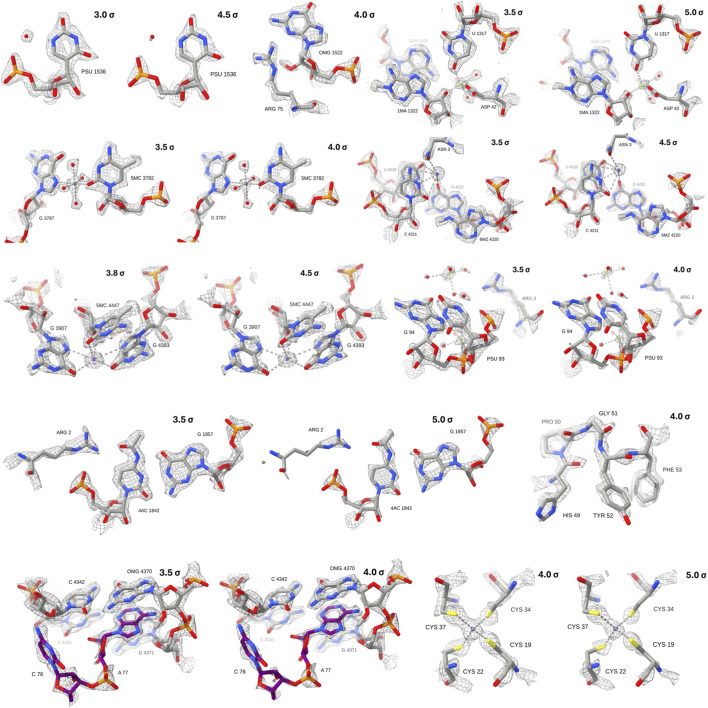
Model-in-map quality and validation of modified residues. Selected regions of the cryo-EM reconstruction are shown to illustrate the fit of the atomic model into the experimental density. The grey mesh represents the EM map, contoured at the σ levels indicated in the top right of each panel.

High-quality cryo-EM density enabled accurate atomic modelling of both ribosomal RNA (rRNA) and protein (rProtein) components. Clear density for rRNA bases permitted unambiguous backbone tracing and base placement, while side-chain density for many rProteins allowed confident modelling of secondary structural elements and amino acid side chains (Figure 7). The close correspondence between experimental maps and fitted models further validates the structural integrity of ribosomes purified using this method.

### Advantages of the protocol

A key advantage of this protocol is the complete omission of translation inhibitors, which are commonly used during ribosome purification but are known to bind conserved functional sites and induce conformational changes. The ability to obtain high-resolution structures without antibiotics indicates that native ribosomal states can be preserved through rapid processing and optimized ionic conditions.

The protocol is compatible with a wide range of limited and sensitive cell types, including pluripotent stem cells and primary immune cells. It relies on standard biochemical reagents and ultracentrifugation equipment available in most structural biology laboratories. The modular nature of the protocol allows individual steps, such as gradient composition or ionic conditions, to be readily adapted for specific applications, including isolation of subunits or ribosome-associated complexes.

## Limitations

The primary limitation of the protocol is the requirement for relatively large numbers of hES cells, which may not be feasible in all experimental settings. Additionally, in the absence of translation inhibitors, ribosome run-off may occur if processing is delayed or if temperature control is insufficient. Strict adherence to rapid, cold handling is therefore essential. Centrifugation times are partly dictated by rotor choice and sample volume; users processing smaller inputs may reduce run times by using smaller rotors without altering buffer composition.

High magnesium concentrations used to stabilize ribosomal complexes may cause aggregation and may not be optimal for all downstream applications. Excess detergent during lysis can disrupt ribosomal integrity, and may cause mitochondrial ribosome contamination, whereas insufficient detergent may result in incomplete lysis and highly reduced yield. Aggregation may arise from incomplete resuspension of sucrose-cushion pellets or from residual sucrose contamination.

### Troubleshooting strategies

Low yield can often be addressed by increasing starting cell numbers, ensuring complete lysis without excessive mechanical force, and allowing sufficient time for gentle resuspension of ribosomal pellets. RNA degradation can be minimized by rigorous RNase control, including bentonite-treated sucrose solutions, fresh addition of RNase inhibitors, and maintenance of low temperatures throughout the procedure. Aggregation observed by negative-stain EM can frequently be reduced by optimizing potassium and magnesium concentrations. If ribosome peaks are poorly resolved on gradients, extending centrifugation time or adjusting gradient steepness may improve separation.

## Discussion

In this practical guide, we describe a robust and scalable protocol for the purification of native 80S ribosomes from hES cells without the use of translation antibiotics. By addressing challenges associated with limited starting material and the sensitivity of pluripotent cells, this method expands the experimental toolkit available for structural studies of human translation under near-physiological conditions.

A defining feature of the protocol is the avoidance of translation inhibitors, which are widely employed to stabilize ribosomes but are known to induce structural and functional artifacts. The successful determination of near-atomic resolution cryo-EM structures from antibiotic-free preparations demonstrates that rapid handling and optimized buffer conditions are sufficient to preserve ribosome integrity. This enables examination of ribosomal conformations that may more closely reflect native cellular states.

Ribosome purification strategies for structural studies have historically relied on similar physicochemical principles, most commonly involving ultracentrifugation through sucrose cushions and gradients. The advance presented here does not lie in introducing a new separation principle, but in demonstrating that an entirely inhibitor-free workflow can be robustly applied to sensitive, non-transformed human cell types while remaining fully compatible with near-atomic resolution cryo-EM. In contrast to most human ribosome preparations, which employ translation inhibitors to stabilize ribosomes during purification, our protocol preserves native ribosomes without drug-induced arrest and yields reproducible high-resolution structures from human embryonic stem cells and primary B cells. Recent low-input affinity-based approaches provide valuable complementary capabilities, particularly when material is limiting; however, these methods typically rely on indirect RNA capture (Erath et al., 2025). Our approach therefore addresses an important methodological gap by prioritizing inhibitor-free preservation of native ribosome structure and functional state heterogeneity in developmentally relevant human cells.

Importantly, analysis of translational state heterogeneity revealed that ribosomes purified under inhibitor-free conditions predominantly occupy non-rotated conformations and lack stable bound tRNA or translation factors, consistent with sampling a near-equilibrium ribosome pool rather than drug-arrested intermediates. While we cannot fully exclude some contribution from ribosome run-off during lysis, rapid harvesting under ice-cold conditions and optimized buffer compositions were used to minimize this effect. Notably, when the same purification strategy was applied to various human cell types, distinct distributions of ribosomal states were observed. This finding indicates that translational state composition is sensitive to cellular context and is preserved rather than homogenized by the purification procedure. The consistent presence of this pool across replicates suggests that it largely reflects a physiologically relevant equilibrium between translating and non-translating ribosomes. This near-equilibrium population likely represents ribosomes poised for initiation or recycling, providing insight into the dynamic distribution of ribosomal states under native, inhibitor-free conditions. Thus, the absence of inhibitors not only avoids structural artefacts but also enables the detection of biologically meaningful differences in ribosome populations across cell types.

The application of the protocol to hES cells is particularly significant, as the majority of existing human ribosome structures have been derived from transformed or cancer cell lines. Ribosomes from pluripotent cells may differ in composition, modification patterns, or conformational dynamics, and the approach described here provides a foundation for investigating such differences at the structural level. While rRNA modification patterns may differ in pluripotent cells, this study does not directly map modifications and future studies will highlight these cell specific patterns. The successful extension of the workflow to primary B cells and iPS cells further underscores its versatility and broad applicability.

While the method requires substantial cell input and careful execution to prevent ribosome run-off, these limitations are balanced by the advantages of preserving native ribosomal architecture and avoiding drug-induced perturbations. Future adaptations may focus on reducing input requirements, isolating specific ribosome populations, or coupling the protocol with functional assays to correlate structure with translational activity.

In summary, this antibiotic-free ribosome purification strategy enables high-resolution structural analysis of ribosomes from human embryonic stem cells and other sensitive mammalian cell types. Its adoption will facilitate future studies of translation regulation in development, differentiation, and disease, and support comparative analyses of ribosome structure across diverse cellular contexts.

## Data Availability

The original contributions presented in the study are included in the article/supplementary material, further inquiries can be directed to the corresponding author.
